# Th1-Mediated Immunity against *Helicobacter pylori* Can Compensate for Lack of Th17 Cells and Can Protect Mice in the Absence of Immunization

**DOI:** 10.1371/journal.pone.0069384

**Published:** 2013-07-11

**Authors:** Hua Ding, John G. Nedrud, Thomas G. Blanchard, Brandon M. Zagorski, Guanghui Li, Jessica Shiu, Jinghua Xu, Steven J. Czinn

**Affiliations:** 1 Department of Pediatrics, University of Maryland School of Medicine, Baltimore, Maryland, United States of America; 2 Department of Pathology, Case Western Reserve University, School of Medicine, Cleveland, Ohio, United States of America; 3 Institute for Clinical Evaluative Sciences, Toronto, Ontario, Canada; Veterans Affairs Medical Center (111D), United States of America

## Abstract

*Helicobacter pylori* (*H. pylori*) infection can be significantly reduced by immunization in mice. Th17 cells play an essential role in the protective immune response. Th1 immunity has also been demonstrated to play a role in the protective immune response and can compensate in the absence of IL-17. To further address the potential of Th1 immunity, we investigated the efficacy of immunization in mice deficient in IL-23p19, a cytokine that promotes Th17 cell development. We also examined the course of Helicobacter infection in unimmunized mice treated with Th1 promoting cytokine IL-12. C57BL/6, IL-12 p35 KO, and IL-23 p19 KO mice were immunized and challenged with *H. pylori*. Protective immunity was evaluated by CFU determination and QPCR on gastric biopsies. Gastric and splenic IL-17 and IFNγ levels were determined by PCR or by ELISA. Balb/c mice were infected with *H. felis* and treated with IL-12 therapy and the resulting gastric bacterial load and inflammatory response were assessed by histologic evaluation. Vaccine induced reductions in bacterial load that were comparable to wild type mice were observed in both IL-12 p35 and IL-23 p19 KO mice. In the absence of IL-23 p19, IL-17 levels remained low but IFNγ levels increased significantly in both immunized challenged and unimmunized/challenged mice. Additionally, treatment of *H. felis*-infected Balb/c mice with IL-12 resulted in increased gastric inflammation and the eradication of bacteria in most mice. These data suggest that Th1 immunity can compensate for the lack of IL-23 mediated Th17 responses, and that protective Th1 immunity can be induced in the absence of immunization through cytokine therapy of the infected host.

## Introduction

The association of gastric *Helicobacter pylori* (*H. pylori*) infection with peptic ulcer disease [1] and gastric cancer[2], [3], [4], combined with a world wide prevalence of infection of over 50%, has prompted many laboratories and companies to pursue development of a vaccine[5], [6]. Such a vaccine would have great utility in areas of the world where the incidence of gastric cancer remains high[7]. The majority of prototype *H. pylori* vaccine work has been performed in murine models. These models have been instrumental in identifying potential antigens, delivery routes, and adjuvants[6]. These strategies however, have been unsuccessful when applied to human subjects in clinical trials[5]. Therefore, the mouse model continues to be used to develop improved adjuvants that might be applied in mucosal vaccination strategies in humans, but also to identify the protective immune responses that might be enhanced by vaccine design to achieve improved efficacy in the clinic.

Vaccine induced protection against *H. pylori* or *H. felis* is associated with a significant increase in the gastric inflammatory response,[8], [9] and most likely the participation of granulocytes (PMN)[10], [11]. This inflammation is dependent on CD4^+^ T cells[12], and many studies demonstrate that Th1, Th17, or both Th1 and Th17 immunity are required[10], [13], [14], [15], [16], [17], [18], [19]. Initial studies using mice deficient in the IL-12 family p40 subunit showed a lack of vaccine induced protection[18], [19]. The p40 subunit however is common to IL-12 and IL-23, thus important for the development of both IFNγ mediated Th1, and IL-17 mediated Th17 responses, respectively. Studies to test the importance of Th1 mediated immune responses have provided conflicting data. Whereas several laboratories have employed IFNγ deficient knockout (KO) mice and achieved protective immunity against gastric Helicobacter infection comparable to wild type mice[19], [20], others showed that although immunized IFNγ KO mice achieved a significant reduction in bacterial load compared to nonimmunized control KO mice, this protection was significantly reduced compared to immunization of wild type mice[16], [18]. It was also demonstrated that, unlike T cells from wild type donors, cells from IFNγ KO mice failed to provide protection when adoptively transferred into immunodeficient recipient mice infected with *H. felis*.

The failure of Th1 mediated immunity to adequately account for protective immunity, in addition to the demonstrated requirement for granulocytes in eradicating Helicobacter in models of vaccine-induced immunity[10], [11], has led to increased interest in the potential role of the IL-23/Th17 pathway of immunity. IL-17 is present in biopsies of *H. pylori*-infected patients and mice, suggesting that IL-17 contributes to Helicobacter-associated pathology[21], [22], [23]. Two recent studies employing the IL-23 p19 KO mouse model have demonstrated a reduction in gastric pathology compared to wild type mice during chronic *H. pylori* infection[24], [25]. Our own observations demonstrate a significant increase of IL-17 following challenge of immunized mice, and a strong Th17 recall response in the T cells from immunized mice[10].

Two laboratories have treated immunized mice with IL-17 neutralizing antibody during *H. pylori* challenge and significantly reduced vaccine efficacy[14], [17]. Conversely, we have used IL-17 KO mice and IL-17R KO mice to demonstrate that in the absence of Th17 cells vaccine induced protective immunity is comparable to immunized wild type mice.[13] These two approaches differ in that in one model the mouse responds to vaccination in the absence of Th17 cells, and in the other the Th17 cell is prevented from performing its effector function after immunity has already been induced. These studies indicate that Th17 cells do indeed play an important role in the protective immune response to *H. pylori*, but that when absent during vaccination, compensatory mechanisms are expanded that make up for the absence of Th17 cells. To further explore the potential of the host to overcome the absence of Th17 cells in the development of *H. pylori* immunity, we used IL-12 p35 KO and IL-23 p19 KO mice to assess the efficacy of vaccination.

We now demonstrate that deletions in either the p35 or p19 subunits that result in IL-12 and IL-23 deficiency respectively, may compromise the protective immune response but that bacterial loads remain significantly reduced. Neither deficiency results in a complete loss of vaccine efficacy. Mice lacking in IL-23 developed a T memory response with a significantly increased Th1 component. In the context of previously published reports, these data suggest that when the host has a congenital lack of the IL-23/Th-17 axis, it develops compensatory mechanisms that mask the importance of Th-17 cells in providing vaccine-induced protective immunity in the wild type host. We further demonstrate that in the absence of immunization, cytokine therapy can promote a Th1 response capable of eradicating Helicobacter from the gastric mucosa.

## Materials and Methods

### Ethics Statement

This study was carried out in strict accordance with the recommendations in the Guide for the Care and Use of Laboratory Animals of the National Institutes of Health. The protocols were approved by the Institutional Animal Care and Use Committees of the University of Maryland in Baltimore and Case Western Reserve University. All efforts were made to minimize pain and suffering.

### Bacteria

*H. pylori* Sydney strain 1 (HpSS1)[26] was grown on Columbia blood agar plates plus antibiotics (7% horse blood (Cleveland Scientific, Bath, OH), 20 µg/ml trimethoprim, 16 µg/ml cefsulodin, 6 µg/ml vancomycin, and 2.5 µg/ml amphotericin B (Sigma, St. Louis, MO) at 37°C for 4–5 days under microaerophilic conditions as previously described[10]. *H. felis*, previously isolated from a domestic cat stomach by our laboratory was grown under similar conditions but substituting polymixin B (0.125 µg/ml) for cefsulodin[27]. *H. pylori* was transferred to Brucella broth containing 10% FBS and antibiotics and grown at 37°C and 10% CO_2_ in preparation for infection of mice. *H. felis* was recovered from agar plates and diluted in PBS.

### Mice

Wild type BALB/c, C57BL/6, and C57BL/6-*il12a^tmlJm^* (IL-12 p35 KO) mice were purchased from The Jackson Laboratory (Bar Harbor, ME). IL-23p19 KO mice were a generous gift of Dr. Nico Ghilardi (Genentech, Inc., South San Francisco, CA). All mice were housed, and all transgenic gene deficient knockout strains were bred, under pathogen-free conditions in microisolator cages at either the Case Western Reserve University or University of Maryland Baltimore Animal Resource Center. This study was carried out in strict accordance with the recommendations in the Guide for the Care and Use of Laboratory Animals of the National Institutes of Health. The protocols were approved by the Institutional Animal Care and Use Committee at Case Western Reserve University and the University of Maryland in Baltimore. All efforts were made to minimize pain and suffering.

### Immunization and challenge

Groups consisted of 6 to 8 week old naïve, unimmunized/challenged (U/C), and immunized/challenged (I/C) mice. Immunized mice received 100 µg HpSS1 plus 5 µg cholera toxin adjuvant (Sigma) in 20 µl PBS by intranasal administration once a week for 4 weeks. Lysate antigens were prepared by sonication and filtration as previously described[10]. Mice were challenged with HpSS1 (approximately 10^7^ CFU) in 0.5 ml Brucella broth by oral gavage 14 days following the final immunization. For treatment of mice with anti-p40 antibody (Cone C17.8, Bio-X-Cell, West Lebanon, NH), mice received 0.5 mg/mouse i.p. injections of antibody on the day of challenge and then at 7 days post-challenge.

### IL-12 therapy

BALB/c mice were infected with approximately 10^7^ *H. felis* organisms on day 1. Half of the infected mice, as well as a group of naïve mice, received daily high dose injections of 1.0 µg IL-12/mouse in PBS i.p. from day −7 to day 7. From day 8 through day 21 mice received a low dose of 0.2 µg IL-12 three times per week. Mice were rested for weeks 4, 5, and 6, given low dose therapy for weeks 7, 8, and 9, and then harvested at the end of week 12. Stomachs were removed and assessed for histologic inflammation in H&E stained sections as described below. Sections were also scored for bacterial load by quantifying the number of infected glands per millimeter length of gastric mucosa.

### Bacterial load determination

**Figure 1:**
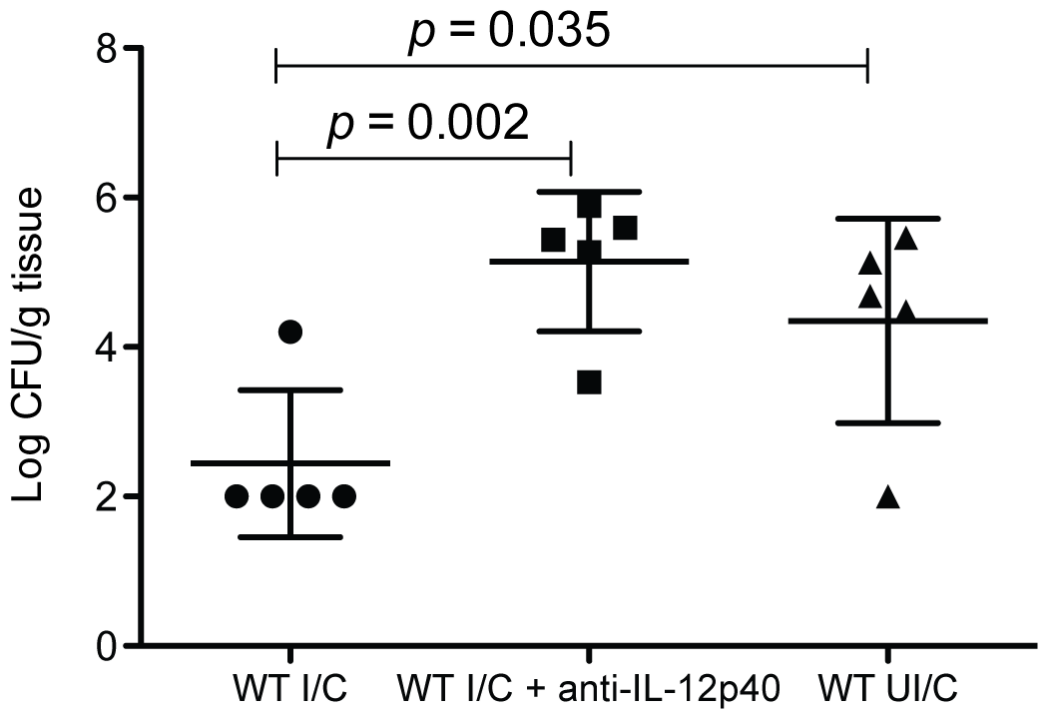
Neutralization of IL-12 and IL-23 reduces immunity in immunized mice. WT C57BL/6 mice were intranasally immunized once a week for four weeks with 100 µg of *H. pylori* sonicate plus 5 µg of cholera toxin. On day14 post-immunization, immunized mice, as well as unimmunized control mice were challenged by orogastric gavage with 1×10^7^ CFU *H. pylori*. Half of the immunized mice received anti-p40 therapy for seven days post challenge. Gastric biopsies were harvested from all mice four weeks post-challenge and evaluated for bacterial load by CFU determination. n = 5 mice/group.

**Figure 2:**
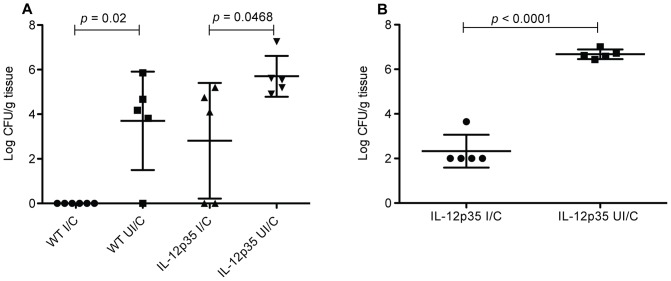
Immunization is protective in mice lacking IL-12. (a) Female WT C57BL/6 and IL-12p35 KO mice, or (b) WT C57BL/6 male mice, were intranasally immunized once a week for four weeks with 100 µg of *H. felis* sonicate plus 5 µg of cholera toxin. On day 14 post-immunization, immunized mice, as well as unimmunized control mice were challenged by orogastric gavage with 1×10^7^ CFU *H. pylori*. Gastric biopsies were harvested from all mice four weeks post-challenge and evaluated for bacterial load by CFU determination. n = 5–6 mice/group.

Mice were harvested at 28 days post-challenge. Stomach biopsies were taken for bacterial load quantification, histologic evaluation, and RNA isolation for cytokine measurements. *H. pylori* bacterial load was determined by colony forming units (CFU) for assessment of p40 neutralization (Figure 1), and for initial experiments testing immunization of IL-12 p35 KO mice (Figure 2). For CFU determination, a 2 mm wide longitudinal strip of the greater curvature of each stomach was placed into pre-weighed 1.5 ml microcentrifuge tubes containing 200 µl urease test broth, weighed again, and then homogenized with disposable pellet pestles (Kontes, Vineland, NJ). Homogenate was diluted serially in 10-fold dilutions in sterile PBS to 1∶1000 and 10 µl of each dilution was plated as described above. Colonies were counted after 5 to 7 days of incubation, and representative colonies were tested for urease, oxidase and catalase activities.

**Figure 3:**
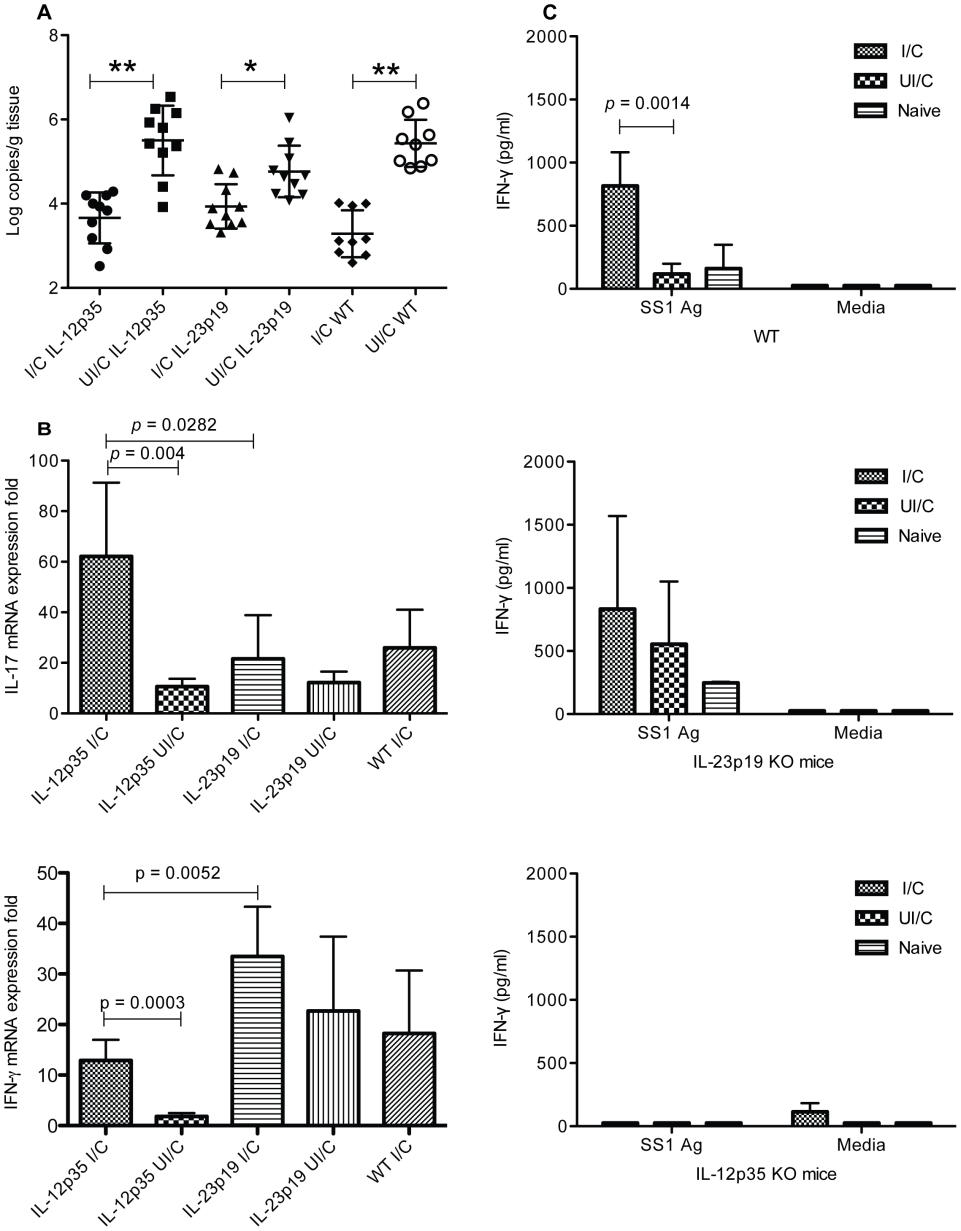
Immunization is protective in the absence of either IL-12 or IL-23. WT C57BL/6, IL-12p35 KO, and IL-23p19 KO mice received intranasal immunizations once a week for four weeks with 100 µg of *H. pylori* sonicate plus 5 µg of cholera toxin. and received an orogastric challenge of 1×10^7^ cfu *H. pylori* on day 14 post immunization. Nonimmune mice were infected at the same time as the immunized mice. (a) Gastric biopsies were harvested from all mice four weeks post-challenge and evaluated for bacterial load by quantitative PCR. (b) The remainder of the stomachs were processed for RNA isolation the IL-17 levels were determined by RT-PCR. n = 9–10 mice/group. (c) Splenocytes prepared at harvest from 5 mice per group were stimulated *in vitro* for 48 hours with 10 µg/ml *H. pylori* lysate antigen and the supernatants were evaluated for IFNγ levels by quantitative ELISA.

Partway through these experiments, the gas generating envelope system used for maintaining microaerobic cultures was discontinued by the manufacturer. Therefore, bacterial load determinations for a comprehensive analysis of immunization with IL-12 p35 KO mice and IL-23 p19 KO mice were performed using quantitative PCR (Figure 3). Total DNA was extracted from frozen gastric tissue using DNeasy (Qiagen) but with an additional step following Proteinase K digestion in which the samples were incubated at 95°C for 10 minutes to help release bacterial DNA. PCR amplification was performed on an Eppendorf Realplex real time thermocycler (Westbury, NY) using primers for ureC as previously reported[28] or with primers specific for the *H. pylori* 16s rRNA gene,[29] and a standard curve consisting of purified chromosomal DNA from *H. pylori* SS1. For each sample the PCR reaction was performed in triplicate with the SYBR Green supermix (Fermentas, Glen Burnie, MD). For *H. felis* bacterial load determination, the numbers of *H. felis* infected glands per linear millimeter of antral and fundic mucosa were determined by examination of silver-stained tissue sections.

### Histologic evaluation

A second biopsy was cut along the outer curvature of the stomach, fixed in 10% buffered formalin, and evaluated for histologic gastritis using H&E-stained sections by a pathologist blinded to sample identities. For *H. pylori* infected tissue, the most inflamed area of each section was given a score of 0–5 for the extent, depth, and makeup of cellular infiltrate as well as changes in tissue architecture, as previously described in detail for *H. pylori* infection.[8] For *H. felis* infected tissue the antral and fundic mucosa were each given separate global scores based on the criteria described above.

### Gastric cytokine determination

For quantification of cytokines, total RNA was extracted from frozen gastric tissue using RNeasy (Qiagen) and then RNA (0.5 µg) was converted to cDNA using a reverse transcription kit (Qiagen). PCR amplification was performed using a two step cycle of 95°C for 15 sec. followed by 60°C for one min for 40 cycles in an Eppendorf realplex^2^ Mastercylcer (Hamburg, Germany). Primer sequences were as follows: mIL-17 forward: GCT CCA GAA GGC CCT CAG A, mIL-17 reverse: AGC TTT CCC TCC GCA TTG A [30], IFNγ forward::CATggCTgTTTCTggCTgTTACTg, IFNγ reverse: gTTgCTgATggCCTgATTgTCTTT[16]. Gastric tissue RNA from a naïve mouse in each group was chosen as a calibrator using relative analysis real-time PCR. Fold differences in the expression of genes in the tissue were calculated as 2^(CtGene-CtGAPDH)-(CtGene-CtGAPDH)^.

### Recall assay

Spleens were harvested and single cell suspensions were prepared after lysis of red blood cells. Unfractionated cells were plated at 1.0×10^6^ cells per well in 96-well flat bottom plates in RPMI 1640, 10% FBS media. Cells were stimulated with *H. pylori* SS1 sonicate at the indicated concentration, in triplicate. Concanavalin A (Sigma) stimulation at 1 µg/ml was used as a positive control. Supernatants were collected after 48 hours incubation and the concentration of IFN-γ and IL-17 was determined using a quantitative sandwich ELISA according to manufacturer's instructions (R&D Systems).

### Statistics

Statistics were determined using ANOVA. Differences between groups were considered significant at an interval level of *P*<0.05.

## Results

### Antibody mediated depletion of p40 subunit cytokines during challenge results in loss of protective immunity

The inflammatory pathways involving p40 have been demonstrated to be highly associated with vaccine-induced protective immunity against *H. pylori* as demonstrated through the use of p40 deficient mice[18], [19]. The use of knockout mice results in a loss of IL-12 and IL-23 activity throughout the course of both immunization as well as the challenge phase of these experimental vaccine studies. To determine if IL-12 or IL-23 activity during the immunization phase is sufficient to induce protective immunity, we used antibody mediated depletion of IL-12 and IL-23 in wild type mice through the injection of anti-p40 antibody during the challenge phase. Mouse stomachs were assessed 28 days post challenge by bacterial load determination. There was 1.88 log reduction in CFUs in immunized mice compared to nonimmune control mice (Figure 1; *P* = 0.035). Treatment of immunized mice with anti-IL-12 antibody eliminated protection, resulting in a significantly higher bacterial load than nonantibody treated immunized mice (*P* = 0.002), and no difference compared to nonimmune control mice.

### Immunization of IL-12 p35 deficient mice results in protection against *H. pylori* challenge

Mice deficient in the p35 subunit and therefore lacking IL-12 were used to determine the impact on protective immunity when IL-12 is absent at all stages of the experiment. Wild type C57BL/6 and p35 KO mice were immunized as described above and challenged. Stomachs were evaluated for bacterial load 28 days post challenge. The WT mice displayed a reduction in bacterial load of 3.7 log CFUs compared to nonimmunized mice (Figure 2a; *P* = 0.02). Immunization of p35 KO mice resulted in reduced yet significant immunity with a drop in bacterial load compared to nonimmune p35 KO mice of 2.9 log CFU (*P* = 0.047). It has been reported that mouse gender can be determinative of degree of Helicobacter induced gastric inflammation with female mice responding to infection with more rapid and pronounced inflammation[31]. Female mice also tended to be better protected following immunization with a recombinant Salmonella vector[32]. Since inflammation is believed to participate in the protective immune response, we also assessed the efficacy of vaccination in this model with male mice. Similar to female mice, male p35 KO mice immunized against *H. pylori* had a reduction in bacterial load of 4.34 CFUs compared to nonimmune control p35 KO mice (Figure 2b; P<0.0001).

### Lack of either p35 or p19 does not prevent vaccine induced reductions in bacterial load

IL-12 contributes to the generation of a Th1 based immune response characterized by the production of IFNγ from Th cells. However, Th17 based immunity has recently been shown to play a significant and possibly essential role in the vaccine induced protective immune response to *H. pylori*. Since the generation of Th17 cells is dependent upon IL-23, and therefore the p19 cytokine subunit, we compared p35 and p19 KO mice for their relative ability to develop protective immunity against *H. pylori* challenge. Immunization of WT C57BL/6 mice resulted in a reduction of 2.2 log CFU compared to nonimmune control mice (Figure 3a, *P*<0.0001). Immunization of either p35 or p19 deficient mice also resulted in significant reduction in bacterial load relative to their respective nonimmune controls (*P*<0.0001 and *P* = 0.0137 respectively), but the lack of p19 had a more detrimental impact on protection with a decrease in bacterial load of only 0.9 log CFU compared a decrease of 1.9 log CFU for p35 KO mice. There was no significant difference in the bacterial load of immunized KO and WT mice.

### Immunized mice compensate for lack of IL-12 or IL-23

Gastric tissue was processed for RNA isolation and levels of IL-17 and IFNγ message were measured by quantitative PCR as a measure of Th17 and Th1 cell activity respectively. Immunized/challenged mice deficient in p19 did not produce significantly greater levels of IL-17 than nonimmune control p19 KO mice (Figure 3b). Immunized IL-12 p35 KO mice produced significantly greater levels of IL-17 compared to nonimmune control mice (*P* = 0.004) as well as IL-23 p19 KO (P = 0.0282). Similarly, in IL-23 p19 deficient mice tested for IFNγ, we observed a significant increase in cytokine production in immunized/challenged mice compare to immunized/challenged IL-12 p35 KO mice (Figure 3b lower panel; *P* = 0.0052). There was an increase of IFNγ in immunized/challenged IL-12 p35 KO mice compared to their nonimmunized controls (*P* = 0.0003) indicating that the p35 deficiency did not result in complete reduction of IFNγ. While IL-23 p19 KO mice failed to produce significantly greater amounts if IFNγ than immune WT mice, IFNγ is typically high following immunization of WT mice.

Spleen cells prepared from mice at harvest were stimulated with *H. pylori* lysate antigen and the amount of IFNγ and IL-17 present in the cell culture supernatants at 48 hours were determined. No IL-17 was detected in either the nonimmunized challenged or immune mice (data not shown). Significant levels of IFNγ were present in the supernatants of WT immune mice compared to nonimmune controls (P = 0.0014) as has been reported (Figure 3c)[15]. Conversely, little IFNγ was detected for mice deficient in IL-12 p35 KO mice and no differences were observed between immune and nonimmune groups. Immunized challenged IL-23 p19 KO mice did not produced more IFNγ than the nonimmunized challenged p19 KO mice but both WT and p19 KO immunized challenged mice produced significantly greater amounts of IFNγ than immunized challenged IL-12 p35 KO mice (p<0.05).

### Promotion of Th1 immunity protects mice from chronic Helicobacter infection

**Figure 4:**
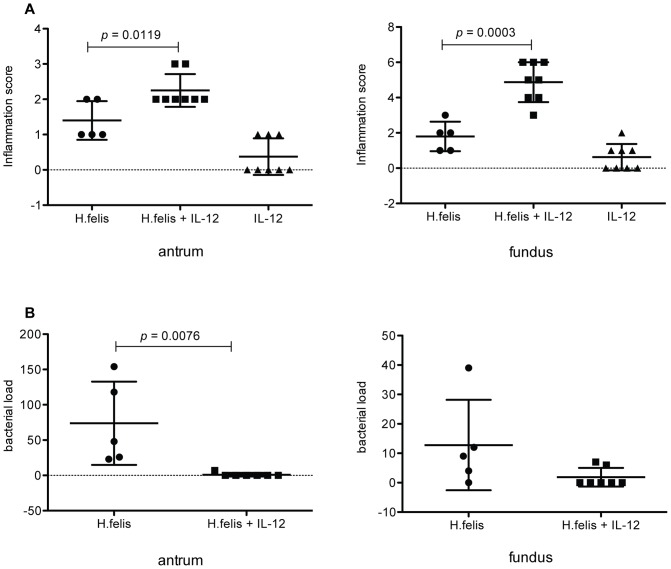
Treatment of *H. felis* infected mice with long term IL-12 therapy is protective. Balb/c mice were infected with *H. felis* and treated with IL-12 intermittently for 12 weeks. Control mice were either infected with *H. felis* without treatment, or treated with IL-12 in the absence of infection. Mice were harvested at 12 weeks and longitudinal biopsies encompassing the entire length of the gastric mucosa were prepared for histologic analysis. (a) H&E stained sections were used to independently grade the antrum and fundus for inflammation using scales of 0–3 and 0–10 respectively. (b) Silver stained sections were used to visualize *H. felis* organisms and bacterial load was determined by counting the number of infected glands per linear millimeter in the antrum and fundus. n = 5–8 mice/group.

Immunized mice deficient in p19 IL-23 remained protected and had elevated levels of IFNγ. To determine if promotion of a strong Th1 response is sufficient to protect the host against chronic Helicobacter infection even in the absence of immunization, BALB/c mice were treated with IL-12 during the course of *H. felis* infection. BALB/c mice were selected because they have been described to develop milder inflammation in response to Helicobacter infection and develop a weaker Th1 response.[33] The ability to significantly increase the type of Helicobacter-associated inflammation that could lower the bacterial load in this model would represent a more stringent test of the therapeutic potential of the cytokine therapy. Analysis of gastric tissue 12 weeks after infection revealed significantly heightened histologic antral and fundic inflammation in *H. felis*-infected mice treated with IL-12 compared to mice receiving either IL-12 alone or infected with *H. felis* without treatment (Figure 4a; P<0.02 and P<0.0003 respectively). IL-12 treatment was effective at reducing the bacterial load of *H. felis* as bacteria could be observed in only two out of seven mice (Figure 4b). Overall reductions in bacterial load were highly significant in the antrum (*P* = 0.0076), although the lower loads generally observed in the fundic mucosa in *H. felis* infected mice resulted in a lack of significance when compared to the infected mice treated with IL-12.

## Discussion

The present study demonstrates that vaccine-induced protective immunity against *H. pylori* can be achieved in hosts deficient in p19 IL-23. Additionally, p19 IL-23 deficient mice, compromised in their ability to generate Th17 cells, respond to immunization and challenge with increased levels of IFNγ producing Th1 cells. These results therefore, support previous studies suggesting that redundant protective anti-*H. pylori* mechanisms exist in the mouse which can cross-compensate for each other's absence[13], [14], [20]. These data also demonstrate that the induction of Th1 immunity through the application of IL-12 can be sufficient to significantly reduce bacterial load and even eradicate Helicobacter organisms from the gastric mucosa in the absence of vaccination.

IL-17 producing cells have been shown to play an essential role in the protective immune response against *H. pylori* in immunized mice[14], [17]. Two independent studies used depletion of IL-17 during challenge of immunized mice to achieve a loss of immunity. These results are consistent with our previous studies documenting the rapid and significant rise in IL-17 in the gastric mucosa following challenge of immunized mice relative to IFNγ [10]. Mice that did not receive immunization prior to challenge failed to demonstrate a rise in IL-17 or IFNγ within the first two weeks following challenge. We also noted a strong in vitro Th17 recall response in the spleen cells of immune mice in that study and demonstrated that protection was dependent upon neutrophils, an aspect of inflammation promoted by IL-17 production.

Our failure to eliminate protection by using p19 IL-23 deficient mice does not conflict with these prior studies demonstrating an essential role for Th17 cells. Rather, our results help illustrate that the timing of the deficiency is crucial in shaping the anti-*H. pylori* response. Indeed, our prior studies using IL-17 and IL-17R deficient mice also demonstrated that in the absence of Th17 cells, vaccine-induced protective immunity can be induced against *H. pylori* [13]. In both the p19 IL-23 and IL-17 deficiency models, the immune response induced by immunization occurred in the absence of Th17 cells and therefore a secondary mechanism capable of providing equivalent protection was induced. Our current studies indicate that IFNγ producing cells are significantly elevated. Conversely, the two studies that achieved depletion of IL-17 through the administering of IL-17-specific blocking antibodies applied the antibodies during the challenge phase. The protective, vaccine-induced Th17 dominant immune response had already developed so that when the recall response was required in vivo to protect against the challenge organisms, the host had not developed a compensatory response.

Similar results have been achieved for IFNγ. Several laboratories observed protective immunity against *H. pylori* comparable to wild type mice when they used IFNγ knockout mice[19], [20]. Although such results cast doubts on the importance of Th1 cells in the vaccine induced protective immune response, others demonstrated significantly reduced protection in IFNγ^−/−^ mice compared to wild type mice[16], [18]. The present data indicate a compensatory IFNγ response is induced in the absence of p19 IL-23, suggesting that Th1 immunity has the potential to protect mice against chronic *H. pylori* infection. These observations have practical implications for *H. pylori* vaccine design in that it is likely that a successful vaccine need not necessarily, specifically induce a strong Th17 response, but alternatively may be efficacious if designed to stimulate Th1 immunity or even a combined Th17/Th1 response.

Our data from *H. felis*-infected mice treated with IL-12 illustrate that Th1 mediated immunity can be protective and that such a response can be achieved even in the absence of immunization. These results are particularly enlightening given our understanding of how Helicobacter infection of the gastric mucosa induces regulatory T cells that limit host immunity. Despite the presence of histologic inflammation, both mice and humans generate Treg cells that dominate the host response[34], [35], [36], [37]. Thus, immunization strategies that to date have achieved little success in clinical trials, and which rarely induce sterilizing immunity in mice, must evolve to overcome the propensity of the host to suppress the response during natural or experimental challenge[5], [6]. The significant reductions in bacterial load, and demonstrated absence of *H. felis* in many of the mice following IL-12 therapy indicate that inducing protective immunity may indeed be an achievable goal, and that supplementation of vaccines with specific cytokines may constitute a strategy for achieving such aims.

*H. felis* was chosen to test the potential benefit of IL-12 therapy for its ease of identification in histologic sections, and for the nature of the inflammation associated with infection [27], [38] **.** Our use of Hp SS1 results in colonization almost exclusively of the antral-fundic junction. The inflammatory response induced by Hp SS1 is also localized to this region of the gastric mucosa. The use of *H. felis* allowed for colonization throughout the antrum and also included the fundus, a pattern of colonization that more accurately reflects human infection by *H. pylori*. This colonization pattern, and the ease with which *H. felis* can be identified by microscopic evaluation, provided a means of assessing the ability of the inflammatory response to eradicate the bacteria in two distinct regions. A similar evaluation for *H. pylori* is impractical in mice since visualizing the bacteria is much more difficult. The inability to discern the antral-fundic border at dissection would make an evaluation as the eradication of bacteria in the two separate regions impossible with tissue-based assays for bacterial load such as quantitative PCR or a determination of CFUs. An evaluation of the efficacy of IL-12 therapy for *H. pylori* in mice may yield similar results to those reported here for *H. felis*. A reduction in efficacy compared to the *H. felis* model however may be difficult to interpret, as it could simply be a reflection of the mild inflammation normally induced by Hp SS1 in mice as opposed to the robust gastritis induced by *H. felis* in mice or by *H. pylori* in humans.

In summary, these results help demonstrate that, although Th17 cells have been demonstrated to be an essential component of the vaccine induced protective immune response in mice, when a host is compromised in the ability to develop Th17 immunity during vaccination, compensatory mechanisms exist that result in the development of immunity equivalent to the wild type host. This immunity is characterized by a recall response the results in elevated levels of IFNγ. The induction of IFNγ producing Th1 immunity can be achieved by the application of IL-12 in the infected host resulting in protective immunity even in the absence of immunization, further demonstrating the protective potential of Th1 cells against gastric Helicobacter infection. These results may aid in the design of new experimental vaccines that achieve improved efficacy.
